# The Establishment of a Novel γ-Interferon In Vitro Release Assay for the Differentiation of *Mycobacterial Bovis*-Infected and BCG-Vaccinated Cattle

**DOI:** 10.3390/vetsci11050198

**Published:** 2024-04-30

**Authors:** Yuhao Zhao, Wentao Fei, Li Yang, Zhijie Xiang, Xi Chen, Yingyu Chen, Changmin Hu, Jianguo Chen, Aizhen Guo

**Affiliations:** 1 National Key Laboratory of Agricultural Microbiology, College of Veterinary Medicine, Huazhong Agricultural University, Wuhan 430070, China; zhaoyuhao@webmail.hzau.edu.cn (Y.Z.); 18871703093@139.com (W.F.); xiangzhijie2024@163.com (Z.X.); chenxi@mail.hzau.edu.cn (X.C.); chenyingyu@mail.hzau.edu.cn (Y.C.); hcm@mail.hzau.edu.cn (C.H.); chenjg@mail.hzau.edu.cn (J.C.); 2 Key Laboratory of Development of Veterinary Diagnostic Products, Ministry of Agriculture and Rural Affairs, Huazhong Agricultural University, Wuhan 430070, China; 3 Hubei Hongshan Laboratory, Huazhong Agricultural University, Wuhan 430070, China; 4 Key Laboratory of Ruminant Bio-Products of Ministry of Agriculture and Rural Affairs, Huazhong Agriculture University, Wuhan 430070, China; 5 Wuhan Keqian Biology Co., Ltd., Wuhan 430206, China; yangli15527183902@163.com

**Keywords:** differentiation of the infected from vaccinated animals (DIVA), bovine tuberculosis (bTB), vaccination, BCG, interferon-gamma (IFN-γ) in vitro release assay (IGRA), differential antigens, regions of difference (RDs)

## Abstract

**Simple Summary:**

Bovine tuberculosis (bTB) is a complex zoonotic disease that poses challenges in its management throughout numerous countries. The potential of Bacille Calmette-Guérin (BCG) vaccination as a preventative measure against bTB is explored by many researchers. However, traditional tuberculin skin test methods cannot differentiate infected from vaccinated animals (DIVA). In this study, we established a novel interferon-gamma in vitro release assay (IGRA) for the bTB DIVA test by expressing a recombinant fusion protein named RCE that contains three differential antigens (Rv3872, CFP-10, and ESAT-6) that are present in virulent *M. bovis* but lacking in BCG genomes. Using a commercial IGRA bTB diagnostic kit based on several RCE stimulators of peripheral blood mononuclear cells as the references for testing 97 cattle, the RCE optimal concentration and cut-off value were measured using the receiver operator curve (ROC). After that, we utilized RCE-IGRA on calves that had received a BCG vaccination to demonstrate that it could be an ideal DIVA method. Therefore, this study is significant for the application of BCG vaccination and the more effective prevention and control of bTB.

**Abstract:**

BCG vaccination is increasingly reconsidered in the effective prevention of bovine tuberculosis (bTB). However, the primary challenge in BCG vaccination for cattle is the lack of a technique for differentiating between infected and vaccinated animals (DIVA). This study aimed to establish a novel DIVA diagnostic test based on an interferon-gamma in vitro release assay (IGRA). The plasmid encoding three differential antigens (Rv3872, CFP-10, and ESAT-6) absent in BCG genes but present in virulent *M. bovis* was previously constructed. Thus, a recombinant protein called RCE (Rv3872, CFP-10, and ESAT-6) was expressed, and an RCE-based DIVA IGRA (RCE-IGRA) was established. The RCE concentration was optimized at 4 μg/mL by evaluating 97 cattle (74 of which were bTB-positive, and 23 were negative) using a commercial IGRA bTB diagnostic kit. Further, 84 cattle were tested in parallel with the RCE-IGRA and commercial PPD-based IGRA (PPD-IGRA), and the results showed a high correlation with a kappa value of 0.83. The study included BCG-vaccinated calves (*n* = 6), bTB-positive cattle (*n* = 6), and bTB-negative non-vaccinated calves (*n* = 6). After 3 months post-vaccination, PPD-IGRA generated positive results in both vaccinated and infected calves. However, RCE-IGRA developed positive results in infected calves but negative results in vaccinated calves. In conclusion, this DIVA method has broad prospects in differentiating BCG vaccination from natural infection to prevent bTB.

## 1. Introduction

Bovine tuberculosis (bTB) is an important zoonotic disease caused primarily by *Mycobacterium bovis* (*M. bovis*), as well as *M. tuberculosis* (*M. tb*) and *M. caprae*. Its impact extends substantially across the global cattle industry and public health, with particular severity noted in low- and middle-income countries (LMICs) [1,2,3]. Since most LMICs do not accurately report on the state of the bTB pandemic, the true number of infected cattle, despite estimates of 50 million, is probably substantially greater [4]. Currently, no legally approved vaccine prevents bTB in cattle [5]. The main strategy for the control of bTB is “Test and Slaughter” [6]. However, its effective implementation is hindered due to the high economic burden in LMICs [7]. Therefore, it is significant to reconsider vaccination for bTB prevention with BCG, which is the attenuated strain of *M. bovis* and a unique vaccine available for human TB prevention [5,8]. Some efforts in both experimental challenge studies and field trials have been made for the evaluation of BCG’s efficacy on bTB control in cattle and wildlife species in several countries, such as New Zealand, Chile, Mexico, and Ethiopia, but the results have been disappointing [4,9,10,11]. The main reasons for the unsatisfactory results could likely be the standards for protective efficacy based on the pathology typical of bTB, such as the occurrence of tubercles and interference with the traditional intradermal tuberculin skin test (TST) used for the detection of natural bTB [4,9,10,12]. Since tuberculosis is a chronic disease, clinical signs, including typical pathological lesions, may be infrequently observed in infected animals and, therefore, cannot be sensitive parameters for evaluating the efficacy of BCG vaccination. Additionally, traditional diagnostic tests like the TST, based on bovine and avian purified protein derivatives (PPD), and PPD-based IGRA, cannot differentiate between natural infection and BCG vaccination [4,13,14]. However, since the specificity and sensitivity of the IGRA in bTB testing are determined via the specific stimulators of peripheral blood mononuclear cells (PBMCs) in whole blood, there is a potential to utilize differential antigens as stimulators in a novel RCE-IGRA. This approach could replace traditional TST and IGRA methods, enabling differentiation between infected and vaccinated animals (DIVA) and facilitating the evaluation of BCG’s efficacy.

Compared to virulent M. tuberculosis, BCG lacks 16 regions of difference (RDs), including RD1-RD16. RD1, present in mycobacterial species within the tuberculosis complex such as *M. bovis*, *M. tuberculosis*, and *M. caprae*, is absent in most BCG strains [3]. ESAT-6, one of the RD1 antigens, is one of the most common antigens in *M. bovis*, and it has been shown to have a certain capability as a DIVA antigen [12]. Additionally, studies on combined RD1 antigens like ESAT-6 and CFP-10 have demonstrated their strong ability to stimulate cellular immune responses, and ESAT-6/CFP-10-based IFN-γ ELISpot was commercialized as a differential diagnosis between *M. tb* infection and BCG vaccination in humans because BCG is widely used in newborn babies [15,16,17].

A similar strategy has been attempted to control bTB in the cattle industry. To increase the sensitivity of the DIVA test in cattle, other antigens in RDs were selected to combine with ESAT-6/CFP-10. Recently, the cocktail of ESAT-6 and CFP-10 with another RD1 antigen, Rv3615c, was used to develop a new skin test DIVA to distinguish between infected and BCG-vaccinated cattle [14]. However, the increase in skin-fold thickness caused by differential antigens was significantly smaller than the conventional PPD TST, making it prone to false judgments. Furthermore, the TST is a time-consuming and labor-intensive method. Cockle et al. [18,19] assessed the immunogenicity of 28 candidate antigens encoded within the RD1, RD2, and RD14 gene regions that are deleted from the genome of BCG Pasteur. Eight highly immunogenic antigens were identified with potential as diagnostic reagents. The antigen Rv3872, in particular, was an immediate candidate in the differential diagnosis since T cells from BCG-vaccinated cattle failed to recognize it. The RD1 antigen Rv3872 protein (PE35) encoded by the Rv3872 gene is a member of the PE protein family, and it plays a key role in the transport system of the ESAT-6-CFP-10 complex [20,21,22]. Studies have demonstrated that the fusion of ESAT-6-CFP-10 and Rv3872 exhibits stronger immunogenicity than ESAT-6-CFP-10 alone, suggesting the potential for developing a DIVA IGRA [23]. Furthermore, if BCG-vaccinated cattle consistently test negatively using the DIVA IGRA, the cattle are considered uninfected cattle within a bTB-contaminated herd. Therefore, the DIVA IGRA could serve as a valuable tool for evaluating BCG’s efficacy in cattle.

Previously, our laboratory developed and commercialized bovine and avian PPD-based IGRA (PPD-IGRA) for bTB detection in China. In addition, the fusion protein RCE was prepared, and its application was confirmed in the serological test of bTB [24]. The current study aimed to develop an RCE-based DIVA IGRA (RCE-IGRA) for BCG vaccination in cattle. Simultaneously, the DIVA IGRA could be applied to evaluate BCG’s efficacy.

## 2. Materials and Methods

### 2.1. Animal Ethics

The animal experiment in this study was designed and conducted strictly in compliance with the Guide for the Care and Use of Laboratory Animals, Hubei Province, China. The protocols were approved by the Ethics Committee of Huazhong Agricultural University (Agreement No. HZAUCA-2022-0002).

### 2.2. Animals

The research site was a commercial dairy farm housing Holstein cattle located in Hubei Province, China. The comparative intradermal TST and a commercial IGRA from Wuhan Keqian Biology Co., Ltd., Wuhan, China, were both performed to evaluate the herd’s bTB status. The optimum concentration and cut-off value of RCE were determined by sampling 74 positive and 23 negative animals in total. Cattle with an unclear history of infections were selected, and sampling was conducted on 86 of the cattle to test their consistency. Meanwhile, 18 cattle were selected for the DIVA to assess the ability of RCE-DIVA.

### 2.3. Sample Collection

Blood samples were obtained from the caudal (tail) or jugular vein of the animals and subsequently collected in vacutainer tubes containing sodium heparin to prevent coagulation. These samples were then preserved at a temperature of approximately 20 °C and promptly transported to the National Animal Tuberculosis Para-Reference Laboratory (Wuhan, China) of the Ministry of Agriculture and Rural Affairs for PPD-IGRA and RCE-IGRA analyses within 18 h following collection.

### 2.4. BCG Growth

The BCG Danish strain 1311 used in this study was provided by Prof. Kanglin Wan from the Chinese Center for Disease Control and Prevention. The BCG vaccine was stationary cultured in 30 mL of Middlebrook 7H9 Broth (BD, Sparks, MD, USA) with an OADC enrichment medium (BD, Sparks, MD, USA) to a mid-log phase (OD_600_ = 0.6) at 37 °C for 12–15 days. Subsequently, it was harvested via centrifugation (3000 rpm for 15 min), and the supernatant was discarded and replaced with HBSS at 1 mg and suspended with 3 mL of HBSS. The bacterial concentration (CFU/mL) was determined retrospectively by plating tenfold dilutions in Middlebrook 7H9 Broth with OADC and counted on 7H11 agar with OADC. The one-milligram moist weight of BCG was equivalent to 2.6–4.3 × 10^6^ CFU.

### 2.5. BCG Vaccination Procedures

Twelve clinically healthy and negative-for-bovine-IFN-γ, under-one-month-old calves were randomly divided into the non-vaccinated control group (*n* = 6) and the BCG vaccination group (*n* = 6). In addition, six one-year-old cattle were selected for the infected group (*n* = 6) after testing positive for bTB using TST and IGRA. The non-vaccinated and bTB-negative group received equivalent doses of HBSS. In contrast, the vaccination group was administered a subcutaneous injection of BCG 10^6^ CFU of 3 mL in the mid-neck. The cattle of the three groups were kept in separate pens. PPD-IGRA and RCE-IGRA were detected in the calves at 90 d after BCG vaccination.

Infected cattle should be defined as follows: OD_630_ PPD-B – OD_630_ PBS ≥ 0.2, OD_630_ PPD-B – OD_630_ PPD-A ≥ 0.2, and OD_630_ RCE – OD_630_ PBS > 0.15.

Immunity-positive (BCG-vaccinated) cattle should be defined as follows: OD_630_ PPD-B – OD_630_ PBS ≥ 0.2, OD_630_ PPD-B – OD_630_ PPD-A ≥ 0.2, and OD_630_ RCE – OD_630_ PBS ≤ 0.15.

bTB-negative cattle should be defined as follows: OD_630_ PPD-B – OD_630_ PBS < 0.2, OD_630_ PPD-B – OD_630_ PPD-A < 0.2, and OD_630_ RCE − OD_630_ PBS ≤ 0.15.

### 2.6. Commercial PPD-IGRA

The blood samples were collected from each cattle in 4 mL of heparinized whole blood for bTB detection using the commercial IGRA kit, following the manufacturer’s instructions (Wuhan Keqian Biology Co., Ltd., Wuhan, China). Subsequently, the blood was transferred to several 2 mL tubes at 1 mL/tube. Then, cellular stimulation was immediately performed above the tubes; bovine PPD (PPD-B) (100 IU), avian PPD (PPD-A) (100 IU), and PBS, each 100 μL, were separately added into tubes with the blood samples, mixed, and incubated at 37 °C for 20 h. Then, the tubes were centrifugated (3000 rpm for 5 min), and the plasma was transferred into new tubes. The plasma IFN-γ concentrations were measured using the commercial sandwich ELISA of the IGRA kit. The bTB-positive or bTB-negative results were determined based on OD_630_ values, according to the manufacturer’s instructions.

### 2.7. Expression and Purification of RCE

The competent *E. coli* BL21 (DE3) cells were electroporated and transformed with recombinant pET-28a-RCE encoding the fused genes Rv3872, CFP-10, and ESAT-6 previously constructed at our laboratory. The RCE (about 35 kDa) was expressed under an induction of 0.4 mM IPTG for 3 h and purified with Ni SepharoseTM 6 Fast Flow (GE Healthcare, Uppsala, Sweden) and checked using 10% SDS-PAGE [25]. The concentration was determined with the Enhanced BCA Protein Assay Kit (Beyotime, Shanghai, China).

### 2.8. Establishment of RCE-DIVA IGRA

A total of 74 positive and 23 negative cattle were sampled to determine the optimal concentration of RCE and the cut-off value of DIVA RCE-IGRA, and the 6 mL heparinized whole blood from each cattle was collected. The RCE concentrations for DIVA RCE-IGRA were optimized with the above commercial PPD-IGRA kit. Briefly, the heparinized whole blood was transferred to different tubes at 1 mL/tube. To each tube was added 100 μL of RCE with various final concentrations of 2 μg/mL, 4 μg/mL, 6 μg/mL, and 8 μg/mL, respectively, as well as 100 μL of PPD-B, PPD-A, and PBS, as instructed for the kit. Then, the tubes were gently shaken to mix the blood and the stimulators, and the tubes were incubated at 37 °C, for 20 h. The supernatant plasma was taken, and IFN-γ was tested with the IGRA kit. The OD_630_ values of RCE-stimulated blood were recorded as OD_RCE_, while the OD_630_ values of PBS-stimulated blood were OD_PBS_. The values of OD_RCE_-OD_PBS_ were calculated, and serials of cut-off values, sensitivities, and specificities were calculated using the conventional PPD-based IGRA as the reference. Then GraphPad Prism 8.0 software was used to make the receiver operating characteristic curve (ROC). The final cut-off value was defined as the difference of OD_RCE_-OD_PBS_ with the biggest area under curve (AUC) corresponding to both the highest sensitivity and specificity with a 95% confidence interval (CI).

### 2.9. Coincidence Rate Evaluation and Correlation Analysis

To evaluate the coincidence rate, a total of 86 cattle with unknown infections were tested in parallel with both RCE-DIVA IGRA and PPD-IGRA kits. Then, all parameters, including the coincidence rate, positive predictive value, negative predictive value, and kappa value with a 95% confidence interval (CI), were calculated. Spearman’s correlation analysis was conducted to determine the relationship between the PPD-IGRA and RCE-DIVA IGRA results.

### 2.10. Statistical Analysis

All parameters were calculated using the online Epitools developed by the Australian Bureau of Animal Health Veterinary Epidemiology (http://www.ausvet.com.au/, accessed on 5 July 2021). The chi-square test was used to detect the statistical difference, and *p* < 0.05 was considered a significant difference and marked as follows: *, *p* < 0.05; **, *p* < 0.01; and ***, *p* < 0.001. Meanwhile, ns represented no significant difference with *p* > 0.05.

## 3. Results

### 3.1. Determination of RCE Optimal Concentration and Cut-off Value for DIVA RCE-IGRA

**Figure A1:**
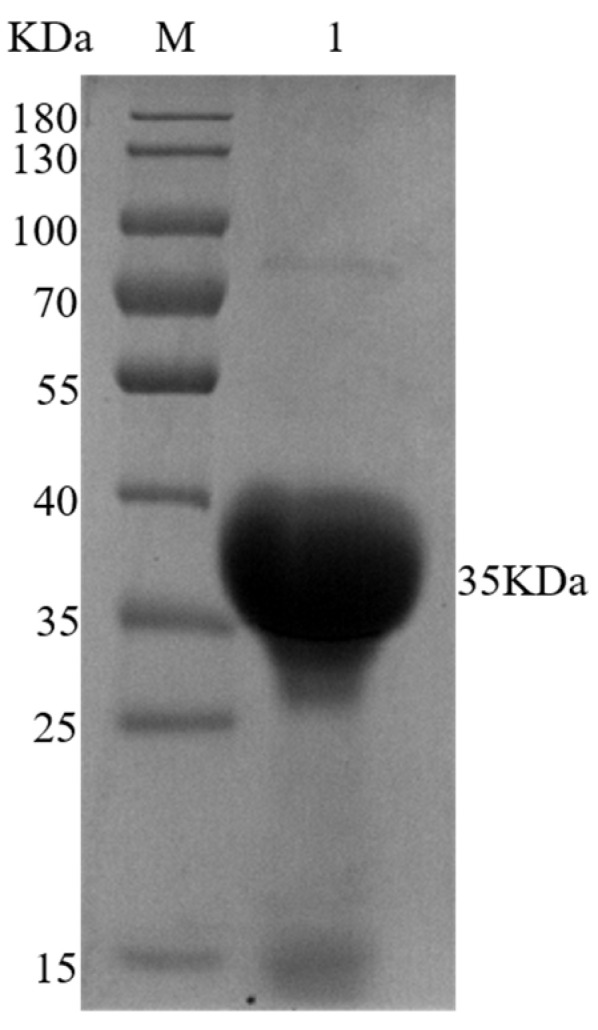
Purified RCE of 35 kDa was checked using 10% SDS-PAGE. Lane M represents the marker of the molecular mass; Lane 1 represents the purified RCE.

The RCE was purified and confirmed with 10% SDS-PAGE (Appendix A Figure A1). The concentration of the stocking solution was 3.0 mg/mL.

**Figure 1:**
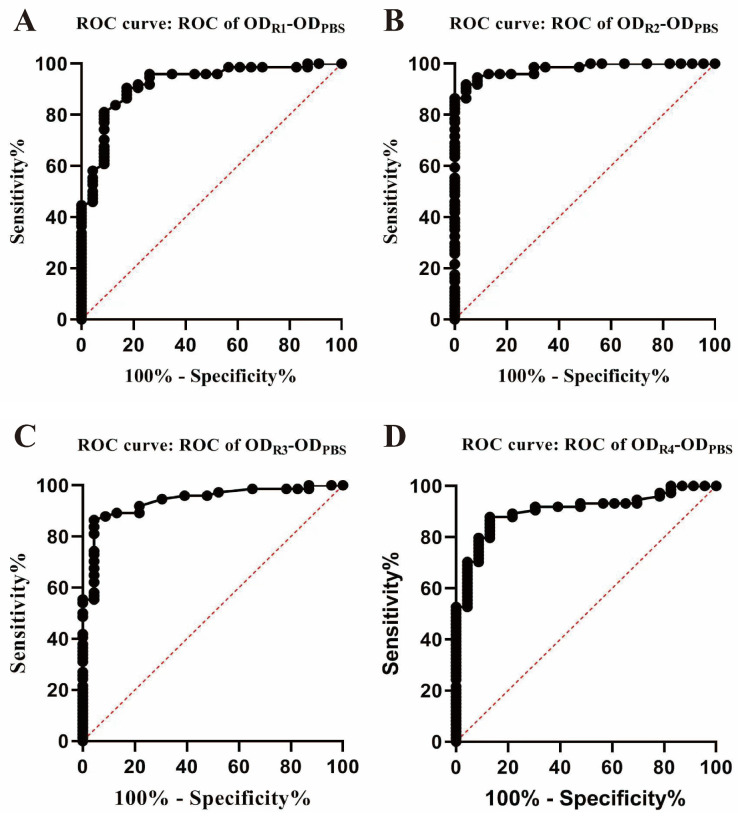
The ROC was used to determine the cut-off value of RCE-DIVA IGRA for bTB diagnosis. (**A**) Stimulation of blood with 2 μg/mL of RCE. (**B**) Stimulation of blood with 4 μg/mL of RCE. (**C**) Stimulation of blood with 6 μg/mL of RCE. (**D**) Stimulation of blood with 8 μg/mL of RCE. The commercial IGRA for bTB diagnosis was used to test 97 cattle blood samples with 74 bTB-positive and 23 bTB-negative cattle as the references.

There were 97 cattle (74 positive and 23 negative cattle) tested using the DIVA RCE-IGRA. Four concentrations of RCE developed four ROCs, and each ROC generated a cut-off value of OD_RCE_-OD_PBS_ (Figure 1). Specifically, when the RCE concentration was set to 2 μg/mL, the cut-off value was 0.09 with 81.08% (60/74, 95% CI: 70.71–88.38) that tested positive and 91.30% (21/23, 95% CI: 73.20–98.45) that tested negative, respectively. When the RCE concentration was set to 4 μg/mL, the cut-off value was 0.15, 91.89% (68/74, 95% CI: 83.42–96.23) that tested positive, 95.65% (22/23, 95% CI: 79.01–99.78) tested negative respectively. When RCE concentration was set to 6 μg/mL, the cut-off value was 0.10 with 86.49% (64/74, 95% CI: 76.88–92.49) that tested positive and 95.65% (22/23, 95% CI: 79.01–99.78) that tested negative, respectively. When the RCE concentration was set to 8 μg/mL, the cut-off value was 0.06 with 87.84% (65/74, 95% CI: 78.47–93.47) that tested positive and 86.96% (20/23, 95% CI: 79.01–99.78) that tested negative, respectively.

When taking the four ROCs together, 4 mg/mL was the ideal concentration of RCE-stimulated whole blood, the cut-off value of OD_RCE_-OD_PBS_ was 0.15, and the corresponding AUC was 0.98 with the diagnostic sensitivity and specificity of 91.89% (95% CI: 83.42–96.23) and 95.65% (95% CI: 79.01–99.78), respectively (Table 1 and Figure 1).

### 3.2. Coincidence Rate Evaluation and Correlation Analysis

The coincidence rate of the commercial PPD-IGRA and RCE-DIVA IGRA is detailed in Table 2. The blood samples of 86 cattle with unknown infection were tested. Compared with the results of commercial PPD-IGRA, the positive coincidence rate was 89.86% (62/69, 95% CI: 80.21–95.82), the negative coincidence rate was 93.20% (96/103, 95% CI: 86.50–97.22), and the total coincidence rate was 91.86% (79/86, 95% CI: 83.95–96.6). The kappa value was 0.83 (95% CI: 0.71–0.95), indicating high consistency between these two methods in bTB detection. When PPD-IGRA was used as the diagnostic reference, the positive predictive value was 93.94% (31/33, 95% CI: 79.77–99.26), and the negative predictive value was 90.57% (48/53, 95% CI: 79.34–96.87).

**Figure 2:**
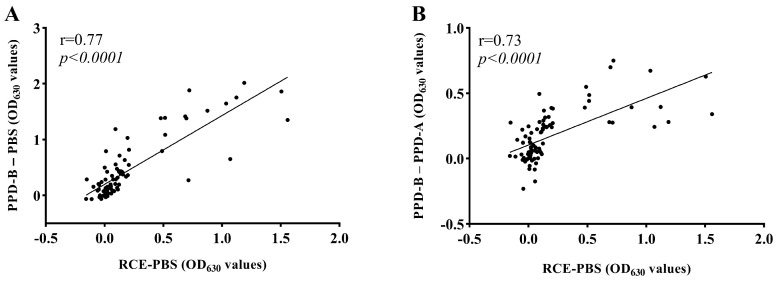
Spearman’s correlation analysis of plasma samples from 86 cattle stimulated with PPD and RCE OD_630_ values. (**A**) The correlation between PPD-B–PBS and RCE-PBS OD_630_ values in the plasma samples. (**B**) The correlation between PPD-B–PPD-A and RCE-PBS OD_630_ values in plasma samples.

Furthermore, we performed Spearman’s correlation analysis of the plasma samples from 86 cattle stimulated with PPD and RCE OD_630_ values. There was a strong positive correlation between PPD-B and RCE OD_630_ values in plasma samples from the same animals (r = 0.77 and *p* < 0.0001, Figure 2A) with the PPD-B–PPD-A and RCE OD_630_ values (r = 0.73, *p* < 0.0001, Figure 2B).

### 3.3. Differential Diagnosis of BCG-Vaccinated and Naturally Infected Calves

**Figure 3:**
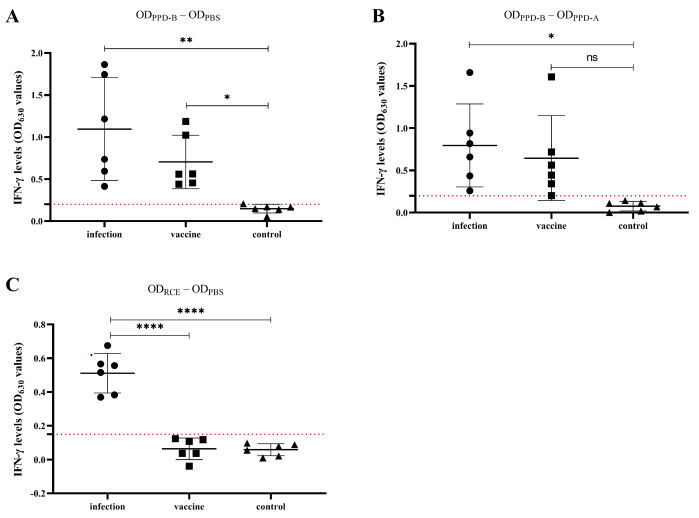
Differentiation of naturally infected and BCG-vaccinated calves via RCE and PPD-based whole blood IFN-γ release assay (IGRA). (**A**,**B**) IFN-γ levels of calves in infected, vaccinated, and PBS control groups detected via commercial PPD-IGRA (the red dashed line represents the cut-off value of 0.20). (**C**) IFN-γ levels of calves in infected, vaccinated, and PBS control groups detected via RCE-IGRA (the red dashed line represents the cut-off value of 0.15). ns, not significant. *, **, and **** represent *p* < 0.05, *p* < 0.01, and *p* < 0.0001, respectively.

Three groups of cattle blood samples (4 mL) were obtained from the caudal (tail) or jugular vein at 90 days after BCG vaccination and detected in parallel with both PPD- and RCE-IGRA. The results showed that the whole blood of the BCG vaccination cattle stimulated with PPD produced large amounts of IFN-γ, which suggests that cellular immune responses were generated after vaccination. PPD-IGRA can detect a significant increase in IFN-γ production in both infected and vaccinated calves, and no IFN-γ production was detected in the control group (Figure 3A,B). However, RCE-IGRA detected IFN-γ production of significance only in cattle that were infected, not in vaccinated calves whose IFN-γ levels were below the threshold (*p* < 0.001) (Figure 3C). This indicates that RCE-IGRA can be an ideal DIVA for BCG vaccination.

## 4. Discussion

The test–slaughter policy has been a long-standing global strategy for controlling and eradicating bTB [26,27]. So far, only a few developed countries and some regions/zones in developing countries have obtained official TB-free status, indicating the poor efficiency of this policy [28]. Despite numerous factors that may impede its successful implementation, the primary social obstacle remains the economic burden associated with it [29]. The related biological risk factors include the very high proportion of persistence and chronic pathogenesis of bTB, high prevalence, unclear base number, inefficient restriction of animal movement, and low levels of biosecurity measures at farms in most developing countries [30,31,32]. However, the unique technique measure of the test–slaughter policy is the key issue contributing to all the above factors.

Can we try other approaches to complement the current test–slaughter policy? The answer is yes. For example, in the UK, the incidence of badger TB has decreased year by year since BCG vaccination was used in wild badgers in 2010 [33,34]. However, the major hindrance to implementing BCG vaccination is the failure of traditional TST to differentiate naturally infected bTB from BCG-vaccinated animals [35]. Subsequently, a contradictory or disappointing conclusion on the efficacy of BCG vaccination in some studies was obtained [8,12,15] that originated from the techniques and criteria used to evaluate the protection of BCG vaccination. It would be understandable to obtain unsatisfactory protection results for BCG vaccination against a chronic disease like bTB using conventional parameters such as bacterial isolation and typical pathological lesions. Hence, the solution to these problems lies in developing a DIVA approach for clinical detection and BCG efficacy evaluation, which effectively addresses the limitations of existing methodologies.

Over the past few decades, substantial advancements have been achieved in the field of tuberculosis-specific antigen development. With the completion of the genome sequencing of *Mycobacterium tuberculosis*, *M. bovis,* and BCG, many promising DIVA antigens have been identified using comparative genomics and transcriptome analysis techniques, e.g., Rv3615c, Rv3871, Rv3872, Rv3873, etc., but further validation is needed [36]. ESAT-6 and CFP-10 are the major DIVA antigens, and their sensitivity and specificity can be improved by adding RD antigens to cattle [37].

RCE is a recombinant fusion protein of ESAT-6-CFP-10 and Rv3872. These three proteins belong to RD1 of *M.tb*, and they are present in virulent Mycobacterium tuberculosis complex (MTBC) but absent in BCG; therefore, they have the potential to differentiate between a natural infection of bTB and BCG vaccination. In this study, we stimulated whole blood from cattle with different RCE concentrations for which the basis was already known and determined the optimal concentration of RCE using an ROC curve of 4 μg/mL. Then, we established the DIVA RCE-IGRA and confirmed that this method has a high diagnostic sensitivity of 91.89% (95% CI: 83.42–96.23) and a diagnostic specify 95.65% (95% CI: 79.01–99.78) by comparing it with commercial PPD-IGRA in the detection of bTB. Moreover, the coincidence rate and correlation with commercial PPD-IGRA kits are very high. Furthermore, to investigate whether RCE-IGRA can differentiate effectively between BCG-vaccinated and naturally infected bTB cattle, we vaccinated calves with BCG and used both commercial PPD-IGRA and this homemade RCE-IGRA for detection. The findings verified that this RCE-IGRA can efficiently differentiate BCG vaccination from natural infection in bTB-positive cattle. In addition, if we use the re-infection of MTBC as the criterion to evaluate BCG protection, this RCE-IGRA could be used in the evaluation of BCG efficacy to replace the conventional methods addressing pathological or bacterial evidence. Therefore, by using this RCE-IGRA, BCG vaccination in cattle would not interfere with the current test–slaughter policy but would constitute an efficient complementary approach to prevent and control bTB.

Generally, eradication programs for some important infectious diseases are divided into several stages, such as brucellosis in Australia [38], Mycoplasma mycoides subsp. mycoides SC (contagious bovine pleuropneumonia) [39], and pig pseudorabies in the USA [40], etc. Vaccination is usually used to reduce the prevalence in an epidemic with high prevalence at an initial stage. As long as the condition’s prevalence is reduced below some baseline, such as 5%, vaccination will be inhibited and replaced with a test–slaughter policy. If BCG vaccination and this DIVA RCE-IGRA or other DIVA are cooperatively applied, a similar strategy could be used in bTB control and eradication. However, limitations arose in this study, such as the small size of samples from BCG-vaccinated cattle and differences in the ages of cattle between groups, which may influence immune responses [26].

## 5. Conclusions

In conclusion, with the progressive implementation of the End TB strategy led by the World Health Organization and the failure of the test–slaughter policy for bTB eradication in many countries and regions, BCG vaccination is increasingly reconsidered for the prevention of bTB. The major obstacle to BCG vaccination in cattle is the shortage of a method for DIVA based on the conventional TST. This study developed a DIVA RCE-IGRA and confirmed that it could be used to differentiate naturally infected bTB from BCG-vaccinated cattle, providing support for BCG vaccination against bTB. Additionally, the RCE-IGRA offers a novel approach to assessing the efficacy of BCG vaccination in cattle.

## Figures and Tables

**Table 1:** RCE stimulation concentrations and their corresponding cut-off values, sensitivities (Se), specificities (Sp), and AUCs were calculated using ROCs.

	RCE Concentrations (μg/mL)							
	2		4		6		8	
Cut-off value	0.09		0.15		0.10		0.06	
AUCs	0.92		0.98		0.94		0.90	
Results (No.)	+ve	−ve	+ve	−ve	+ve	−ve	+ve	−ve
	60	21	68	22	64	22	65	20
Se% (95% CI)	81.08 (70.71–88.38)		91.89 (83.42–96.23)		86.49 (76.88–92.49)		87.84 (78.47–93.47)	
Sp% (95% CI)	91.30 (73.20–98.45)		95.65 (79.01–99.78)		95.65 (79.01–99.78)		86.96 (67.87–95.46)	

**Table 2:** Comparison of detection results for PPD-IGRA and RCE-IGRA.

		PPD-IGRA		
		+ve	−ve	Total
RCE-IGRA	+ve	31	2	33
	-ve	5	48	53
	Total	36	50	86
Kappa (95% CI)	0.83 (0.71, 0.95)			

## Data Availability

All data during the current study are available within the article.

## Appendix A

Determination of RCE optimal concentration and cut-off values.

The purified RCE was checked with 10% SDS-PAGE and expressed as a 35 kDa protein. The stock concentration was 3.0 mg/mL (Figure A1).
